# Intertruncal versus classical approach to the ultrasound-guided supraclavicular brachial plexus block for upper extremity surgery: study protocol for a randomized non-inferiority trial

**DOI:** 10.1186/s13063-022-06029-x

**Published:** 2022-01-29

**Authors:** Quehua Luo, Yujing Cai, Hanbin Xie, Guoliang Sun, Jianqiang Guan, Yi Zhu, Weifeng Yao, Haihua Shu

**Affiliations:** 1 Department of Anesthesiology, Guangdong Provincial People’s Hospital, Guangdong Academy of Medical Sciences, Guangzhou, Guangdong 510080 People’s Republic of China; 2 Department of Anesthesiology, the Third Affiliated Hospital of Sun Yat-Sen University, Guangzhou, Guangdong 510630 People’s Republic of China

**Keywords:** Intertruncal approach, Ultrasound, Brachial plexus block, Supraclavicular, Double-injection technique

## Abstract

**Background:**

Ultrasound-guided intertruncal approach (IA) to the supraclavicular block (SB) is recently proposed as a new approach for local anesthetic (LA) injection in terms of the classical approach (CA) at the level of the first rib. The CA-SB has been proven to result in satisfying sensorimotor block, but associate with a high risk of intraneural injection. The aim of this randomized non-inferiority study is to explore whether IA-SB can obtain similar block dynamics, as the CA-SB, but avoiding an intraneural injection during the whole nerve block procedure.

**Methods:**

The total 122 patients undergoing elective upper extremity surgery will be randomly allocated to receive either an IA-SB or a CA-SB using a double-injection (DI) technique. In the IA-SB group, a portion of LA (15 mL) is injected accurately to the intertruncal plane between the middle and lower trunks under real-time ultrasound guidance; then, the remaining volume (10 mL) is carefully distributed to the other intertruncal plane between the upper and middle trunks. In the CA-SB group, the DI technique will be carried out as described in Tran’s study. The primary outcome is the percentage of patients with a complete sensory blockade at 20 min with a predefined non-inferiority margin of − 5%. The secondary outcomes include the sensory-motor blockade of all 4 terminal nerves, onset times of the individual nerves within 30 min, block-related variables, and adverse events.

**Discussion:**

The results will provide sensory-motor blockade-related parameters and safety of the ultrasound-guided intertruncal approach to the supraclavicular block, thereby promoting clinical practice.

**Trial registration:**

Chinese Clinical Trial Registry ChiCTR2000040199. Registered on 25 November 2020

**Supplementary Information:**

The online version contains supplementary material available at 10.1186/s13063-022-06029-x.

## Background

The supraclavicular block (SB) is a popular and approved approach to the brachial plexus because of the greater safety due to real-time ultrasound guidance and better block dynamics known as the “spinal anesthesia of the arm” [1, 2]. Compared with its single- or multiple-injection counterpart, a double-injection (DI) technique has been widely used in the classical approach (CA), which is at the level of the trunks and divisions [3–11]. However, all these techniques are associated with a high risk of intraneural injection, which is known as the sub-epineurium or intracluster injection. Recently, Siddiqui et al. [12] have proposed an alternative approach using the DI technique based on the clear identification of the outer boundaries (epineurium) of each trunk (upper, middle, and lower), named the intertruncal approach (IA). The double injection in IA aims to provide satisfying block dynamics of the entire brachial plexus while avoiding intraneural injection, by which local anesthetic (LA) is accurately deposited to adipose tissue planes between the upper and middle, and the middle and lower trunks. Although the DI techniques (CA vs IA) require injection of LA very close to contiguous compartments of the brachial plexus, there are essential differences between the two approaches with respect to the injected location of LA [2, 13]. In addition, different approaches direct toward the same goal for better block dynamics, that is, LA spreads evenly within the entire brachial plexus sheath without any nerve sparing [11].

Given that, the DI technique has been associated with less needle passes and results in satisfying block dynamics for ultrasound-guided CA-SB [7]. However, no clinical study data about block dynamics, including the proportion of patients with complete sensory-motor blockade and onset times, are available for IA-SB until now. Therefore, our hypothesis in this study is that the novel IA-SB will obtain comparable block dynamics when compared with CA-SB; meanwhile, it is a promising method of placing the LA immediately adjacent to the trunks without the risk of intraneural injection.

## Methods/design

### Trial design and setting

**Fig. 1:**
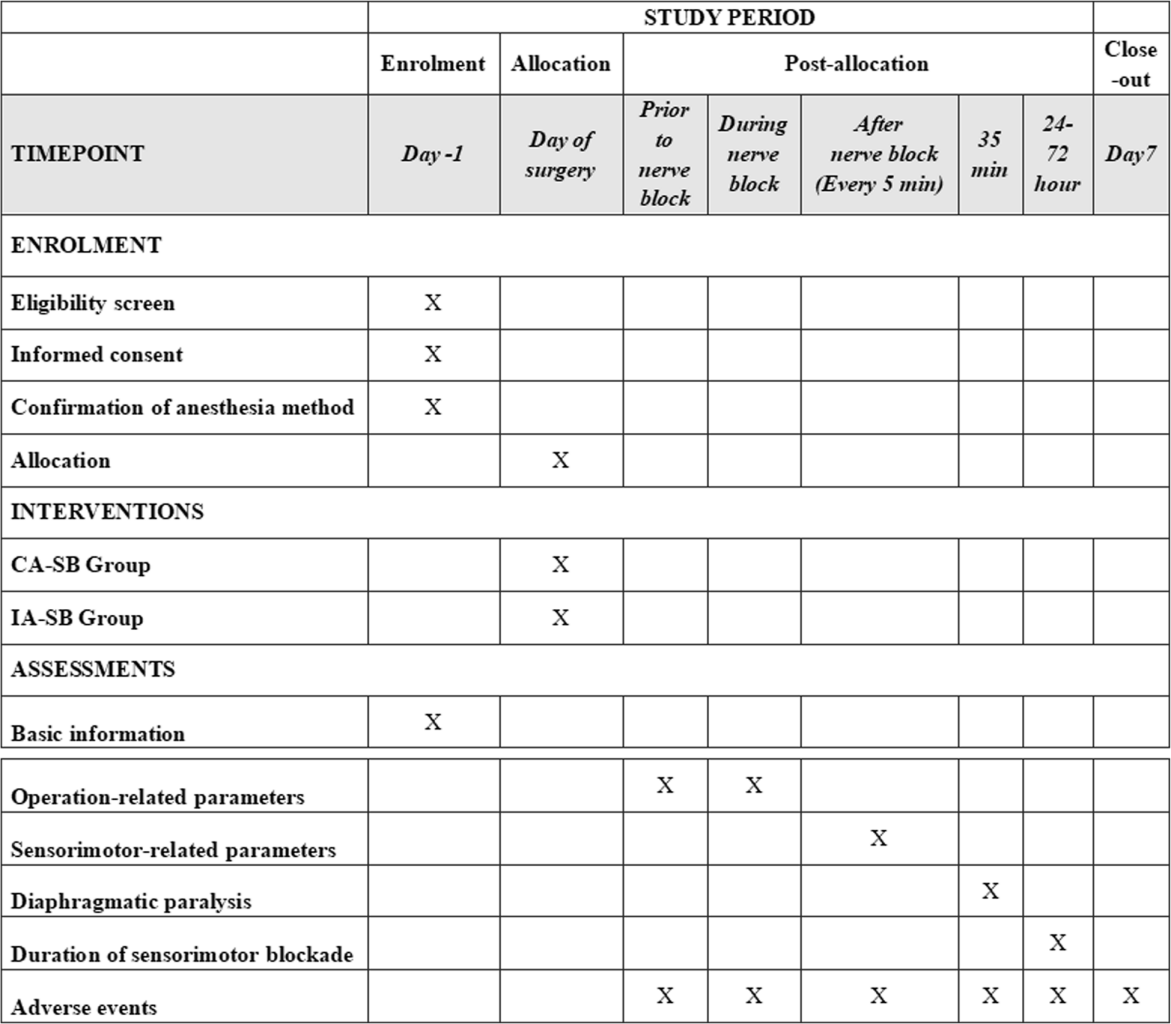
The standard protocol items of this study

**Fig. 2:**
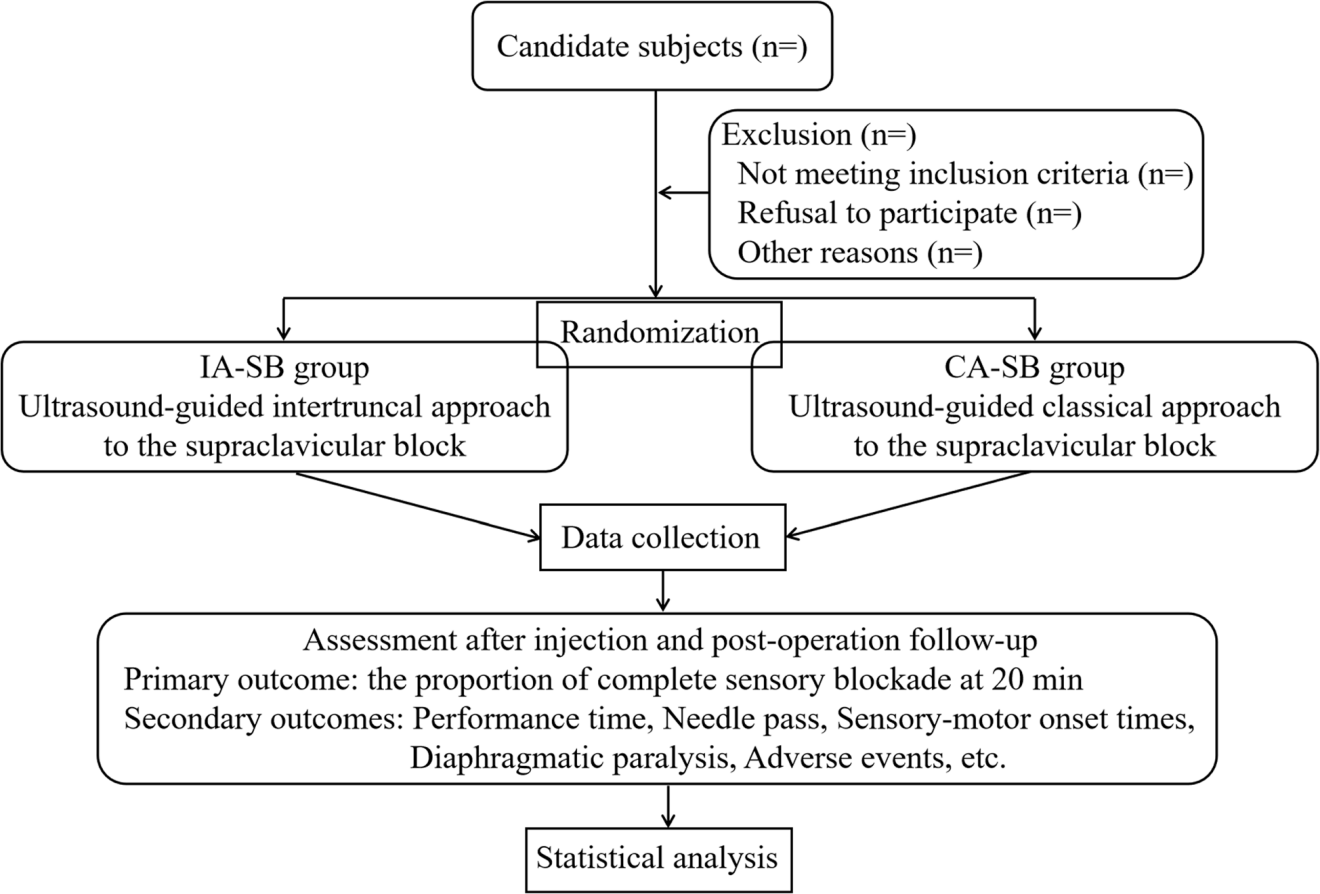
Flow diagram of this study

This prospective, non-inferiority, parallel randomized controlled trial will be carried out at the Third Affiliated Hospital of Sun Yat-sen University, China. It is developed according to the Recommendations for Interventional Trials (SPIRIT) 2013 Statements that came from the Standard Protocol Items (Fig. 1, the SPIRIT Checklist is available as Additional file 1) [14]. Besides, the Consolidated Standards of Reporting Trials (CONSORT) flow diagram will be performed in the design period and completing the study to the end. A detailed flowchart of the trial design is shown in Fig. 2.

### Informed consent

Written informed consent will be obtained from each patient before enrollment. The ultrasound-guided IA- or CA-SB-related operation sequence, benefits, risks, and data privacy of this study will be explained in detail for the participants during the preoperative visit. We will emphasize that this study is voluntary, and the participants are free to withdraw from the study at any time. If the patient is not willing to participate in the study, he or she will receive other means of brachial plexus block or general anesthesia depending on the patient’s willingness and discretion of the treating anesthesiologist.

### Participants and recruitment

The patients scheduled to undergo surgery of the elbow, forearm, wrist, or hand with ultrasound-guided brachial plexus block will be recruited and screened for eligibility in our medical center. An independent researcher will conduct the recruitment when performing the preoperative visit 1 day before surgery.

Inclusion criteria are as follows:

- Signed written informed consent
- American Society of Anesthesiologists (ASA) physical status I to III
- Age 18 to 75 years old
- Operative site at the elbow, forearm, wrist, or hand

Exclusion criteria are as follows:

- Patient’s refusal of brachial plexus block
- Nerve block cannot be performed due to coagulopathy (defined as any coagulation disorders contraindicated to perform peripheral nerve block), pre-existing neuropathy, infection at the supraclavicular fossa, hypersensitivity, or allergy to LA
- Body mass index (BMI) > 35 kg/m^2^
- Pregnancy, severe mental illness, or cognitive dysfunction (unable to communicate or cooperate)

### Randomization and blinding

After the enrollment, the participants were randomly allocated to one of the two groups (CA-SB or IA-SB group) in a 1:1 ratio by computer-generated simple randomization. A sealed opaque envelope that contained a card (recorded with a random number and subject number) will be opened by a research assistant who will not be involved in other stages of this study. Ultrasound-guided CA-SB or IA-SB will be carried out by one out of our nerve block team (WF. Y, JQ. G, and HB. X) and supervised by the coauthor (QH. L), in which all have extensive experience with both techniques (over 60 attempts/per technique) before this study. Another anesthesiologist who is blinded to the randomized allocation and intervention will be responsible for recording the research-related variables and anesthesia management based on the conventional scheme. During the study period, research assistants who will be kept blind to the group allocation oversaw postoperative follow-up by face-to-face assessment or telephone. If a serious adverse event (pneumothorax or LA systemic toxicity, etc.) occurs during the nerve block, un-blinding will be permissible, and then emergency measure will be initiated under the supervision of the outcome assessor.

### Ultrasound-guided techniques

All participants will be seen on 1 day before surgery and demonstrated on the use of a 3-point scale for evaluating sensory-motor blockade. On arrival to the operating room, standard ASA monitors (non-invasive cuff blood pressure, pulse oxygen saturation, and electrocardiogram) and supplemental oxygen (nasal cannula at 4 L/min) will be applied. An intravenous access (20-gauge) for fluid infusion will be established in the contralateral forearm, and the premedication (midazolam 0.05 mg/kg or combined with fentanyl 0.5 μg/kg) will be given prior to nerve block. Drugs that improve the block effect or duration of the sensory-motor blockade will be not allowed to use in the perioperative period, including dexmedetomidine or dexamethasone or magnesium sulfate. All patients will be received EtCO_2_ in monitoring during procedure and surgery. For the two approaches, the nerve block will be performed following standard skin disinfection with a portable ultrasound machine (Sonosite M-turbo, SonoSite, Inc., Bothell, WA) and 80-mm short-beveled stimulating needle (B. Braun Melsungen AG, Melsungen, Germany).

The ultrasound-guided CA-SB with the DI technique will be performed in accordance with the method described in Tran’s study [3]. After obtaining a satisfactory image of elliptical hypoechoic trunks and divisions at the supraclavicular fossa, the operators initially orientate the needle tip to the “corner pocket” between the subclavian artery and the lower trunk with the in-plane technique. A part of the LA (15 mL) of 1:1 mixture of 2% lidocaine (Shandong Hualu Pharmaceutical Co., Ltd.) and 1% ropivacaine (Astrazeneca Pharmaceutical Co., Ltd.) will be injected after the accurate position is confirmed by the “water separation” technique under ultrasound guidance. Subsequently, the needle will withdraw and targets the center of the main neural cluster floated upward by the former LA. The remaining volume (10 mL) will be carefully administered into that central position.

For the ultrasound-guided IA-SB, the procedures with the DI technique are replicated from Siddiqui’s study [12] and the optimal order of injections will be followed according to the suggestion in Endersby’s letter [15]. Using a high-frequency pattern, a consecutive scan will be performed initially at the supraclavicular fossa toward the base of the neck in a coronal oblique plane. Once the three trunks of the plexus (upper, middle, and lower) and its epineurium are well defined, the needle is advanced from the lateral end of the probe, and the first part of LA (15 mL) will be accurately injected into the intertruncal plane between the middle and lower trunks. It is worth noting that each trunk is in a different stage of its trajectory and those divisions have not been fused with each other yet in this area. Then, the second placement of LA (10 mL) is carefully distributed to the other intertruncal plane between the upper and middle trunks.

### Outcome definitions and evaluations

The definitions and evaluations of the primary and secondary outcomes of this study are summarized in Table 1.

**Table 1:** Outcome definitions and evaluations

	Definition	Evaluation
**Primary outcome**		
The percentages of patients with a complete sensory blockade at 20 min	The combined score is equal to or greater than 7 points.	The sensory blockade of the 4 terminal branches of the brachial plexus is evaluated and graded every 5 min until 30 min after injection using a validated 3-point scale*.
**Most important secondary outcomes**		
Nerve injury	The percentages of patients who have been associated with persistent paresthesia or weak of the operative upper limb.	7 days after surgery via post-operation follow-up at the ward or telephone follow-up.
Incidence of adverse events	The percentages of patients who have been occurred with vascular puncture, Horner syndrome, toxicity of LA, or pneumothorax.	According to drawing with blood, real-time ultrasonic image, and (or) the patient’s symptoms. A detailed description is seen in the footnote^£^.
Diaphragmatic paralysis	The excursion of the ipsilateral hemidiaphragm will be measured by ultrasound in supine position via the anterior subcostal route in centimeters.	Using ultrasonic evaluation at 35 min after injection.
**Other secondary outcomes**		
Imaging time	Defined as the time from initial contact of the ultrasound probe with the skin until obtaining satisfactory imaging.	A stopwatch to calculate
Needle time	Defined as the time from initial needle insertion until the complete injection of the LA.	A stopwatch to calculate
Performance time	Defined as the imaging time plus the needle time.	/
Satisfactory imaging	Defined as more than 4 divisions of the plexus should be visualized as hypoechoic circular structures lateral to the subclavian artery for the CA-SB, and all 3 trunks of the plexus (upper, middle, and lower) for IA-SB.	Real-time ultrasonic image
Needle pass	Defined as at least 10-mm withdrawal of the needle to retract its trajectory.	During puncture
Needle visual score	Converted into a 5-point scale^§^	Assessment will be made at the time when the first proper position for LA injection is confirmed.
Procedural-related pain	Converted into the numeric rating scale (NRS)	NRS: 0 = no pain, 10 = worst possible pain
Difficult level	Converted into the NRS	NRS: 0 = no difficulty, 10 = extremely difficulty
Surgical anesthesia	Defined as one can tolerate surgical stimulus	During skin incision
The percentages of patients with a complete sensory or motor blockade	The combined score is equal to or greater than 7 points.	The percentages of patients with complete sensory or motor blockade using a validated 3-point scale^#^ every 5 min until 30 min after injection.
Sensory onset time	The time point when combined score is equal to or greater than 7 points.	Using a validated 3-point scale every 5 min until 30 min after injection.
Motor onset time	The time point when combined score is equal to or greater than 7 points.	Using a validated 3-point scale every 5 min until 30 min after injection.
Sensory-motor onset time	The time point when the combined score is equal to or greater than 14 points.	Using a validated 3-point scale for complete sensory-motor blockade every 5 min until 30 min after injection.
Duration of the sensory-motor blockade	Converted into the NRS	0 = normal compare that to the contralateral upper limb, 10 = no feeling or complete in-mobility; sensory-motor blockade return to normal will be defined as NRS<3.

*3-point scale for sensory blockade: 0 = no block, 1 = partial anesthesia, 2 = complete anesthesia

^£^Vascular puncture is defined as the needle is placed at the optimal location and withdraws with blood before injection under ultrasound guidance. Horner syndrome consisted of the occurrence of symptoms of miosis, ptosis, and hyperemia observed by anesthesiologists within 30 min after injection. Toxicity of LA consisted of the occurrence of signs of agitated, paresthesia, tinnitus, vertigo and perioral numbness, and convulsions, etc., due to central nervous system toxicity observed by anesthesiologists within 30 min after injection. Pneumothorax will be assessed by chest X-ray when the needle accidentally punctures the pleura during performing the block, or the patient feels ipsilateral chest pain, and/or is accompanied by symptoms of severe chest tightness or dyspnea

^§^5-point scale for needle visual score: 1 = very poor, 2 = poor, 3 = fair, 4 = good, and 5 = very good

^#^3-point scale for motor blockade: 0 = no block, 1 = paresis, and 2 = paralysis

### Sample size calculation and statistical analysis

Our working hypothesis is that ultrasound-guided CA- versus IA-SB yield similar block dynamics. Thus, this study will be designed as a non-inferiority trial. In the previous studies using single or multiple injections in SB, we have observed that the proportion of patients with complete sensory blockade reached a plateau starting at 20 min after injection. It fluctuates between 70% and close to 100% within 30 min [3–6, 16]. In other words, relative to itself, the variation is very subtle from 20 to 30 min. The primary outcome is considered as the proportion of patients with complete sensory blockade of all 4 terminal nerves at 20 min after injection in this study. Based on a pilot study with 15 patients in each group, the proportion of patients with complete sensory blockade achieved was 73% in the CA-SB group and 87% in the IA-SB group (unpublished data). Therefore, we assume that a difference in proportion between the two groups less than − 5%, measured at 20 min after injection, will be considered non-inferiority. The required sample size per group is calculated to be 55 with a statistical power of 80% and a one-sided 95% confidence interval. To account for a possible 10% dropout rate, the total sample size is inflated to 122 participants (*n* = 61, per group).

Statistical analysis will be performed using *SPSS for Windows 18.0* (SPSS Inc., Chicago, IL). For continuous data, normality will be first assessed with the *Kolmogorov-Smirnov test* and then analyzed using an *independent-samples t test.* Categorical variables will be summarized as a frequency, *n* (%), such as the proportion of complete sensory or motor blockade, success rates, and adverse events, etc. The *Pearson χ*^*2*^ *test*, *Fisher’s exact test*, or a *Mann-Whitney U test* will be used for categorical variables as appropriate. A *p < 0.05* will be considered statistically significant for all results.

### Data collection and retention

The nerve block-related parameters and postoperative follow-up data will be recorded by a research assistant, and a statistics analysis will be carried out by an independent statistician. To enable examination and re-analysis from regulatory authorities, all electronic data will be desensitized and stored securely at the Department of Anesthesiology of the Third Affiliated Hospital of Sun Yat-sen University for 5 years. Preserved paper materials of this study include the original signed informed consents, study protocol and interventions, and case report forms. The project will be monitored by a data monitoring committee composed of specialists in ethics, anesthesiology, and statistics. These data will be kept in our research database and not revealed to other people without appropriate permission.

### Adverse events

All adverse events will be monitored and recorded. Once any serious adverse event occurs, it will be immediately reported to the research group, which will determine the causality and therapeutic measures of the adverse events. The chief investigator will be responsible for reporting all adverse events to the Ethics Committee.

### Auditing

No formal auditing process is proposed for this trial.

### Protocol amendments

In principle, the established study protocol is not to be modified. Any amendments to the study will be first initiated by the principal investigators and then agreed and confirmed by all study participants. Finally, the modified version of the protocol will be submitted to the Ethics Committee for approval.

### Trial dissemination

The research results and findings will be disseminated in a peer-reviewed journal or at scientific conferences.

### Patient and public involvement

No patients were involved in the design, recruitment, and conduct of the study and were also directly consulted in the development of the research question or outcome measures. An original article will be prepared to present the trial results at the proper time after the end of the study. Results of the final study will be disseminated to all study participants through E-mail recorded at the time of enrolment. The burden of intervention will not be taken by the participants themselves.

## Discussion

The supraclavicular brachial plexus block does gain popularity benefited from the application of ultrasonic visualization technology [17, 18]. However, the optimal needling technique for the SB is remaining controversial. Various studies have proposed a series of techniques of where to first inject the LA and how many injections to perform around the brachial plexus, which include single-, double-, triple-, targeted intracluster-, or modified-injection techniques for the last nearly two decades [3–6, 9, 11, 16]. Up to now, the most common techniques for LA injection described at the first rib level are a single or double injection. The needle intentionally breaches the layer located at the outer border of the entire plexus, and the needle tip is observed as it advances to the “corner pocket” (at the intersection of the first rib and subclavian artery) in real time for a single injection [19]. If a double injection is used, then the needle will be redirected and placed in the middle of hypoechoic structures representing neural tissue [3]. However, these techniques have been proved to be associated with a high risk of intraneural injection. A recent cadaver study has reported that sub-perineural injections can be as high as 24% for a single intracluster injection [20]. As a result, it should be remembered that it cannot exclude direct or indirect nerve injuries even under real-time imaging of the needle-nerve distance [21, 22]. Therefore, in clinical practice, it is eager to carry out a scheme to prevent nerve injuries and finding a more appropriate injection site will be better and safer according to the needs of inexperienced operators.

The development of a new-style approach or needling technique is coming into clinical practice because the advancement in ultrasound guidance allowed for a further understanding of the underlying anatomy of the brachial plexus (trajectory and surrounding fascial sheath) [23]. For instance, the selective trunk block which is a novel brachial plexus block technique is just inspired by accurately identifying the main components of the plexus above the clavicle under real-time visualization and is expected to result in producing surgical anesthesia of the whole upper extremity [24, 25]. Recently, Siddiqui and colleagues [12] have proposed an alternative approach to the classical approach to a SB, in which LA is carefully injected into the investing adipose layers between the trunks. This is a very promising needling technique without intraneural injection for ultrasound-guided SB. However, the specific data of block dynamics is not yet clear compared with the classical approach. The objective of this study was to assess the block dynamics and clinical feasibility with DI in the supraclavicular fossa via IA.

In the previous technique-related trials, the more needle passes seem the shorter onset time and better sensory-motor blockade [7]. However, the shorter onset time associated with the incremental needle passes is counterbalanced by its longer performance time and is accompanied with a high risk of intraneural injection [3–6, 16]. Nonetheless, the most used and teaching needling technique is the DI technique because, with an acquired satisfactory learning curve, the trainees can implement relatively safe skills while retaining the benefit of quick onset [26, 27]. In addition, we also observed that, in many patients, rather than being a singular entity, the neural cluster is composed of more than one main satellite clusters after the first injection [6, 8]. Finding a better approach for better LA diffusion based on the advancement in ultrasound guidance might be a meaningful technological innovation. Excitingly, the novel IA is just performed between the epineurium of the 3 trunks, and it aims to anesthetize the brachial plexus where the 3 trunks can be visualized as independent of hypoechoic nodules [12]. The authors have proposed that this approach can provide complete blockade of all 4 terminal nerves and satisfying onset times while avoiding intraneural injection and pleural puncture. In our study, the sensory-motor blockade-related clinical parameters including the percentage of patients with complete blockade at all the predetermined intervals, onset times, and adverse events will be recorded. As the IA is closer to the interscalene, an important issue concerning the potential risks of diaphragmatic paralysis is another important dimension of safety [28, 29]. The excursion of the ipsilateral hemidiaphragm will be measured by M-mode ultrasonography in supine position via the anterior subcostal route in centimeters at 35 min after injection [30]. We will also observe the duration of sensorimotor block and potential nerve injury after surgery. The results can help us to determine the efficacy and safety of an IA with the DI technique.

In conclusion, this trial should enable us to better assess the effectiveness of an IA to ultrasound-guided SB, with the potential possibility of avoiding intraneural injection. It may provide us with an ideal inserting approach for optimized risk-benefit at the supraclavicular fossa. It should also advance the understanding of the optimal site of injection and optimization of the diffusion of LA in this inserting approach with the DI technique.

## Trial status

The study will be conducted over a period of more than 9 months (from 26 November 2020 to 31 August 2021) with the latest version of the protocol. At the time of manuscript submission, candidates had been included and some patients had participated in the study.

## Supplementary Information

**Additional file 1.** SPIRIT 2013 Checklist.

## Data Availability

All investigators will have access to the final de-identified study dataset from the corresponding author for the purpose of scientific publications.

## Abbreviations

IA: Intertruncal approach
CA: Classical approach
SB: Supraclavicular block
LA: Local anesthetic
DI: Double injection
ASA: American Society of Anesthesiologists
CONSORT: Consolidated Standards of Reporting Trials
SPIRIT: Standard Protocol Items: Recommendations for Interventional Trials
